# Morphology-Mechanical Performance Relationship at the Micrometrical Level within Molded Polypropylene Obtained with Non-Symmetric Mold Temperature Conditioning

**DOI:** 10.3390/polym13030462

**Published:** 2021-01-31

**Authors:** Sara Liparoti, Andrea Sorrentino, Vito Speranza

**Affiliations:** 1 Department of Industrial Engineering, University of Salerno, via Giovanni Paolo II, 132, 84084 Fisciano, Italy; vsperanza@unisa.it; 2 Institute for Polymers, Composites and Biomaterials (IPCB-CNR), Via Previati, 1/C, 23900 Lecco, Italy

**Keywords:** nano-indentation, polymer morphology, mechanical properties, polymer processing

## Abstract

The control of the structural properties of a polymeric material at the micro and nano-metrical scale is strategic to obtaining parts with high performance, durability and free from sudden failures. The characteristic skin-core morphology of injection molded samples is intimately linked to the complex shear flow, pressure and temperature evolutions experienced by the polymer chains during processing. An accurate analysis of this morphology can allow for the assessment of the quality and confidence of the process. Non-symmetric mold temperature conditions are imposed to produce complex morphologies in polypropylene parts. Morphological and micromechanical characterizations of the samples are used to quantify the effects of the processing conditions on the part performance. Asymmetric distribution of temperatures determines asymmetric distribution of both morphology and mechanical properties. The inhomogeneity degree depends on the time that one side of the cavity experiences high temperatures. The spherulites, which cover the thickest of the parts obtained with high temperatures at one cavity side, show smaller values of elastic modulus than the fibrils. When the polymer molecules experience high temperatures for long periods, the solid-diffusion and the partial melting and recrystallization phenomena determine a better structuring of the molecules with a parallel increase of the elastic modulus.

## 1. Introduction

The knowledge of the structural properties of a polymeric material on the micro and nanometric scale is strategic to obtain parts with high performance, durable and free from sudden failures [1,2]. For example, the properties of semi-crystalline polymers depend on the characteristics and the particular combination of the amorphous and crystalline phases formed during the solidification [3,4]. Determining the mechanical properties of the individual phases with traditional techniques is challenging due to the size of the structures [5]. For this reason, micro and nanoscale characterization techniques have established themselves both in the field of scientific research and in the field of industrial research [6]. Some of these techniques are now accessible for accurate characterizations, even at the production stages [7,8]. Among the others, nano-indentation and atomic force microscopy (AFM) based techniques (HarmoniX and Peak Force) are the most interesting and promising [9]. Nano-indentation is a recently developed technique, which differs from traditional hardness tests in the possibility of monitoring the depth of penetration during the loading and unloading phases [10,11,12]. The load-depth of the penetration diagram provides more information than a microscopy image of the residual impression, as it reveals the “history” of elastic and plastic deformation [13]. HarmoniX and peak force AFM techniques are able to simultaneously provide topographic and mechanical maps during the scanning of the sample at the nanometric scale [14,15,16]. With these techniques it is possible to define the mechanical characteristics (hardness and Young’s modulus of different phases, adhesion of a dispersed phase to the polymeric matrix, etc.) of both polymers and composites when small volumes of the test material are available or when it is necessary to have a low depth of penetration. The time-dependent properties generally displayed by polymers limit the usefulness of this characterization in determining their modulus [17,18]. However, this problem has become insignificant for most polymer materials, including most glassy and high crystalline polymers [19].

An interesting application of the micro and nanoscale characterizations concerns the possibility of in-depth study of the effect of the single process parameters on the final characteristics of a polymeric part. This opportunity is particularly interesting for discontinuous transformation processes such as injection and compression molding, rotational molding, blow molding, vacuum forming, et cetera. In this case, complex time-dependent flow fields, temperature and pressure generate intricate morphologies with process specific features [20,21]. A large number of studies on micro and nano characterizations make reference to a variety of polymer materials ranging from highly crystalline polymers [22,23] to low-crystallinity and glassy polymers [24]. However, due to the small size and complexity of the features developed during common processing conditions, an extensive application of these methods requires additional studies.

Injection molded samples show a characteristic skin-core structure characterized by a sinusoidal-type distribution of mechanical modulus [9]. High mechanical modulus is shown by crystalline and oriented structures whereas low values are characteristic of amorphous and low oriented structures [25,26]. The quantity and quality of these structures are strongly dependent on the solidification conditions, which in turn depend on the processing conditions, material properties and mold geometry. When all these aspects are carefully controlled, symmetric morphology distributions with small variations along the thickness are expected. Abrupt geometrical discontinuities and unbalanced mold cooling are common causes affecting part qualities [27,28,29,30]. Incomplete filling, poor surface finishing, uncontrolled part shrinkage and warpage are generally observed [31,32]. Despite previous efforts, a precise correlation between part properties and mold conditions is still not accessible [33,34]. The lack of reliable experimental data is probably the main reason for this weakness.

Some attempts to correlate morphology with the process conditions and mechanical properties have been carried out [9,35]. The findings suggest that both morphology and mechanical properties depend on the temperature and flow fields during the injection molding process [25]. However, the effect of the temperature field has only been accounted for in quiescent conditions, and the effect of flow has only been accounted for qualitatively. This paper has a twofold aim: the characterization at the nanometric scale of injected molded samples, and the correlation of the part characteristics to the flow and temperature fields via simulation. The molded samples, made of a well characterized polypropylene, are produced in different non-symmetrical mold heating conditions, in order to highlight the effect of temperature, also with uneven distribution, and characterized from both morphological and micromechanical point of views. The process has been simulated by commercial software for injection molding and the results of the characterizations have been discussed on the bases of the simulated temperature and flow fields.

## 2. Materials and Methods

The material used for the production of thin-section moldings is an isotactic polypropylene (T30G, Basell, Ferrara, Italy) with a weight-average molecular weight (Mw) of 376,000 and average molecular weight (Mn) of 56,000.

**Figure 1:**
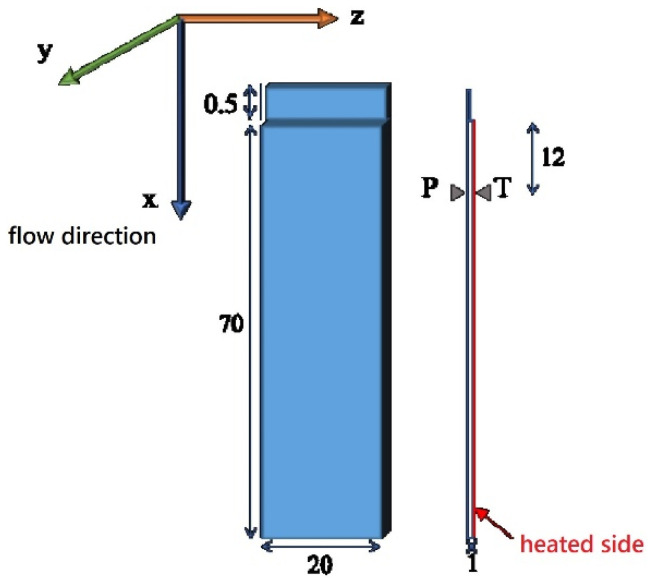
Cavity geometry (drawing units are in millimeters).

Figure 1 shows the dimensions of the test bar and the position of the zones analyzed in a schematic drawing. In particular, the gate thickness is of 0.5 mm and the rectangular cavity has a length of L = 70 mm, width W = 20 mm, and thickness S = 1 mm. The characterizations were conducted 12 mm from the cavity entrance, along the flow-thickness plane.

A 70-ton Negri-Bossi reciprocating screw (Negri Bossi S.p.A., Cologno Monzese, Milano, Italy), injection molding machine was used for the experiments. The injection-molding conditions adopted are summarized in Table 1. Pressure evolution was measured by means of a piezoelectric transducer mounted at the cavity surface. Its location is indicated in Figure 1 as position P. A thin heater layered on the cavity surface was adopted for quick and accurate control of the temperature of one cavity side during the process cycle. Cavity temperature evolution was measured by means of a T-type sensor located on the heater surface. Its location is indicated in Figure 1 as position T.

The tests with cavity temperature modulation were carried out by activating the heater 2 s before the polymer reached position P. After that, the electrical power was held active for an additional heating time, t_h_. During the heating phase, temperature on the heated side of the cavity stabilizes to a constant value reported in Table 1 as cavity temperature of the hot side. A test in conventional injection molding conditions (CIM) was conducted as reference test with the same temperature (25 °C) on both cavity sides.

### 2.1. Optical Microscopy

Slices 0.1 mm thick were cut in position P from injected samples along the flow thickness plane (x,y plane of Figure 1) by means of a Leica slit microtome (model 625, Leica Biosystems, Buccinasco, Milan, Italy). These samples were observed with an optical microscope (model Olympus BX41, Olympus Italia S.R.L., Segrate, Milan, Italy) equipped with a digital camera.

### 2.2. Micro-Indentation

Nano Test Platform (Micro Materials Ltd., Wrexham, UK) was adopted for the characterization of mechanical properties by indentation. The following procedure was adopted: 0.02 mN initial load, 1 mN/s load rate, and 120 mN maximum load with 60 s holding time. A three-sided Berkovich pyramidal diamond tip (100 μm radius) was adopted. The load displacement curves were obtained in different positions along the thickness of the samples. The Oliver and Pharr [36] method was adopted for the evaluation of the elastic modulus. Following that approach, the elastic modulus mainly depends on the initial unloading slope in the load displacement plot.

### 2.3. AFM Analysis

The specimens for AFM investigation were manually cut from the cross section (x,y plane in Figure 1) of the sample in position P and carefully grinded and polished with a predefined schedule in order to prepare the surface for the following tests [35]. A chemical etching, previously adopted on similar iPP samples [35,37] and with 12 h period of etching at room temperature, were carried out in order to remove any residue of the cutting.

NanoScope III (Digital Instruments (DI), Santa Barbara, CA, USA) equipped with HarmoniX tool was adopted for the mechanical and morphological characterization of the samples. Micromechanical and morphology tests were performed by using HMX probe silicon cantilevers (Bruker, Billerica, MA, USA) with nominal radii of ca. 10 nm. Probe calibration was carried out following a procedure reported elsewhere and adopting a standard PS/LDPE sample. The acquisitions were conducted with 0.5 Hz scan rates. NanoScope software version 7.30 and NanoScope Analysis version 1.20 allowed the analysis of each acquisition, in particular the elastic modulus maps were analyzed adopting Derjaguin−Muller−Toporov (DMT) model 38.

## 3. Results

### 3.1. Optical Morphology

**Figure 2:**
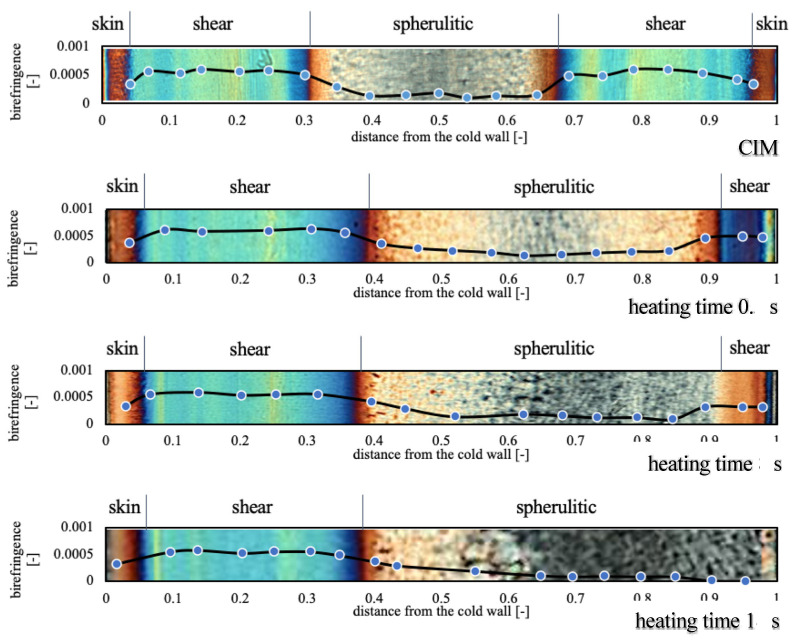
Optical micrographs of the CIM sample and of the samples obtained with 160 °C on the hot cavity side, held for different heating times. Thicknesses of the different layers and birefringence distribution are also reported for each optical micrograph.

Understanding the effects of the molding conditions on the quality of molded parts is important for avoiding long diagnosis and expensive trial and error procedures. To that purpose, a good familiarity with standard sample morphologies is necessary. A representative cross-section morphology showed by the sample produced with a mold temperature of 25 °C is shown in Figure 2. The optical image in polarized light was perfectly symmetric and characterized by alternating colored bands. These interference bands are characteristic of layers with different levels of crystallinity and orientation. The sample layers form at different moments during the injection molding stages, thus, they experience dissimilar thermomechanical histories before their solidification. In the literature, the presence of three distinct morphologies in the molded samples have been reported: a thin, oriented “skin layer” at the sample surface, an oriented non-spherulitic “shear layer” just below the previous one and a less oriented “spherulitic layer” at the sample core [38]. Different mechanisms determine the temperature evolution during the process: fountain flow, heat convection, viscous heating and heat diffusion. Fountain flow allows a continuous mixing of the elements present at the flow front. It holds the temperature profile quite uniform along the thickness at any position during the filling stage [39]. Fountain flow is also responsible for the high level of molecular orientation found at the skin layer [40]. Heat convection and viscous dissipation take place in the sheared fluid layers. In these layers the flow continues to generate molecular orientation with a maximum placed near the already solidified polymer layers at the surface. Soon the temperature profile starts exhibiting a characteristic symmetric profile. In the layers close to the mold wall, the heat diffusion starts exerting an increasing cooling influence, especially when the local flow rate goes down. In the meantime, the sample core is fed with hotter material that tends to increase the temperature differences between the layers. When the flow front goes away, a temperature increase is further aided by the heat generated by viscous flow and crystallization between the core and the skin layers (transition layers). Especially at the cavity center, the packing pressure also contributes to additional molecular orientation and heat generation before gate solidification.

The optical micrographs of the samples obtained by setting the cavity surface temperature at 160 °C for 0.5, 8 and 18 s are shown in Figure 2. In the middle of the cavity close to the hot cavity side, the thicknesses of the different layers are strongly influenced by this external heating. The shear layer thickness undergoes a reduction, whereas the skin layers disappear. On the cold side of the cavity, a small increase in the dimension of the shear layer part that results is pushed in the center molding direction, the sample morphology results are almost unaffected.

Heating for 0.5 s is sufficient to completely destroy the sample symmetry. This can lead to mechanical unbalancing with the formation of warpage and cracks during the service life of the part. High oriented layers are stronger in the direction of flow but weaker in the transverse direction. Also, the shrinkage of the part is highly dependent on the molecular orientation frozen during solidification. A symmetric distribution of the layers in the sample thickness, generally assures stability in the part geometry and uniform response to the external stimuli. Parts with unbalanced morphology can also be subject to surface crazing. It can appear weeks or even months after the part is molded and can be responsible for crack initiation or premature structural degradation [41,42].

The birefringence distributions along the thickness of the samples produced in this work are also reported in Figure 2. The comparison between the birefringence and the morphology images shows that when birefringence is low, the morphology is spherulitic, whereas high birefringence corresponds to fibrillar morphology. Close to the cavity surface, when a low temperature is adopted, a lowering of the birefringence is observed. Birefringence gradually decreases on the heated side with increasing heating time, consistent with the reduction of the shear layer thickness. The birefringence values on the unheated side are more or less the same for all the produced samples.

### 3.2. Elastic Modulus Distribution

**Figure 3:**
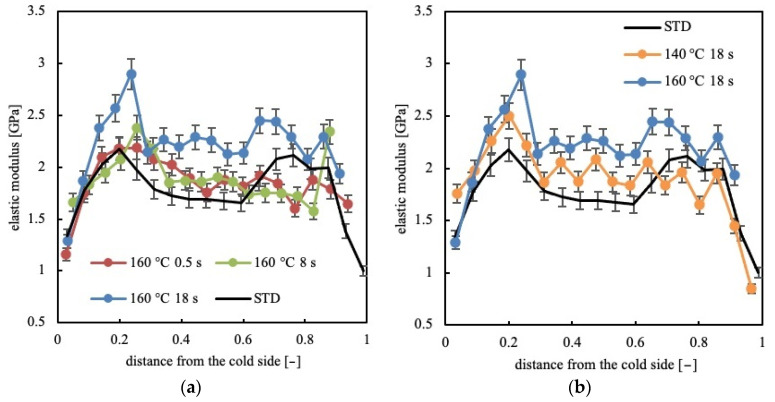
Distribution of the elastic modulus along the sample thickness for the CIM sample and for the samples obtained with different (**a**,**b**) temperatures and heating times on the hot cavity side.

Several nano-indentation tests were carried along the analyzed samples’ thicknesses. Figure 3a shows the distributions of elastic modulus evaluated for the CIM sample and for the samples obtained with 160 °C temperature on the hot cavity side, held for different heating times.

The sample CIM is characterized by the lowest values of the modulus in the skin layers, two maxima in the shear layer, and intermediate values of the modulus in the spherulitic core. The cold sides of the samples 160-05, 160-8 and 160-18 show values of the elastic modulus in the skin layer similar to the values observed in the skin layers of the CIM sample. The increase of temperature on the hot side makes the distribution of the elastic moduli asymmetric. In particular, the elastic modulus on the hot side decreases and the maximum located in the shear layer disappears. On the cold side, the shear layers are characterized by the maximum values of the moduli, which are almost unaffected by the temperature evolution on the hot side, up to 8 s heating time. For longer heating times, 18 s, the elastic modulus shows higher values along the whole sample thickness: the spherulitic core shows values of the elastic modulus that are similar to the values of CIM in the shear layers. Figure 3b shows the distribution of the elastic modulus along the CIM samples, 140-18 and 160-18. Sample 140-18 is composed of fibrillar structures up to 0.4 distance from the cold side, after that, only spherulitical morphology can be detected. In this sample, the spherulitical layer shows values of elastic modulus similar to the values found in the spherulitical core of the CIM sample; the shear layer is characterized by values of the elastic modulus slightly higher than the values found in the shear layer of the CIM sample. Thus, the distribution of the elastic modulus in sample 140-18 is consistent with the distribution of morphology. Sample 160-18 shows a morphology distribution similar to that found in sample 140-18 (see Figure 2); however, the values of the moduli are higher than those found in sample 140-18, especially in the spherulitical layer. It seems that, up to 8 s heating time, the morphology developed along the sample thickness determines the values of the elastic modulus; for heating times longer than 8 s, other phenomena, discussed below, have to determine the values of the elastic modulus.

**Figure 4:**
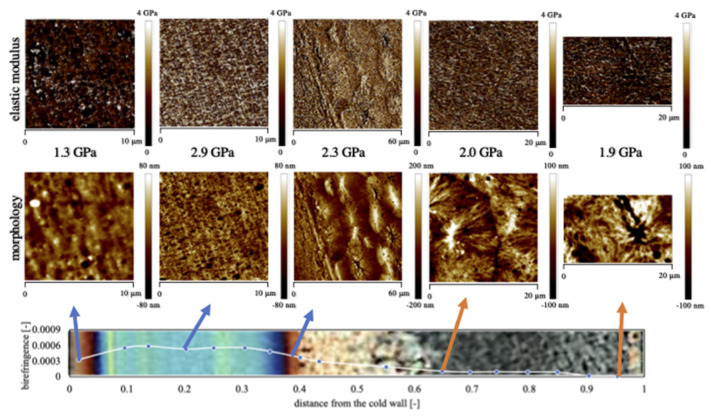
Atomic force microscopy (AFM) acquisition maps of the morphology and elastic modulus related to sample 160-18 in different positions along the sample thickness.

HarmoniX AFM tests supply a complete map of the elastic modulus on the sample surface. DMT models have been applied to the force-indentation curves to obtain the elastic modulus [43]. In this case, a clear correlation between morphology and mechanical properties’ results are more evident. Figure 4 shows the morphology (height) and related map of elastic modulus obtained by the AFM characterizations of the samples produced with 160 °C mold temperature with 18 s heating time.

Figure 4 refers to the morphology observed in different positions along the thickness of a sample obtained with 160 °C cavity temperature on the hot side, held for 18 s. The analyses of the morphological maps on the cold side show that the skin layer region contains oriented and aligned structures. The thickness of this layer depends on the operating conditions and is composed of two areas: one adjacent to the sample wall, composed of elongated structures and another composed by globular elements [44]. The fibrillar morphology developed during the fountain flow is preserved by the fast cooling experienced by the molecules that are in contact with the cold surface of the mold. On the hot side, the morphology of the surface layers is found to be essentially independent of surface heating time, when such a value is lower than 8 s. In that case, the surface layers undergo only some minor changes with time, namely, the number of oriented structures on the sample surface decreases. In samples obtained with 18 s heating time, this morphology is not detectable. In that case, the stretch due to the fountain flow effect has sufficient time to relax. The oriented molecules can return to a quiescent molten state that can subsequently transforms into a spherulitic morphology during cooling at ambient temperature.

By applying external heating, the spherulitic layer enlarges and moves toward the heated surface. The heating of one cavity side probably allows a more efficient stress relaxation and thus a parallel reduction in molecular orientation. This effect is also evident with a short heating time and increases almost proportionally with increase of heating time. With 18 s heating time, the shear layers completely disappear and the spherulitic layer impinges on the sample skin layer. At this temperature, the morphology changes proceed until some critical heating time is reached, above which the transition to the spherulitic morphology occurs.

The elastic modulus maps are also shown in Figure 4. The elastic modulus maps reproduce the same structures shown in the morphology maps; thus, it is possible to recognize the main structures, spherulites and fibrils present along the sample thickness. The average value of the elastic modulus, evaluated by Nanoscope software, is also reported in each map: the elastic modulus is high in the shear layer characterized by fibrils, is very low in the skin layer and intermediate in the spherulitical layer.

**Figure 5:**
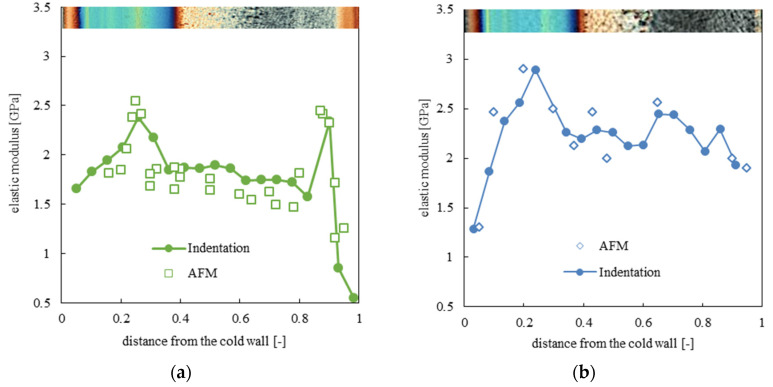
Distribution of the average values of the elastic moduli, evaluated from the AFM maps, along the thickness of samples (**a**) 160-8 and (**b**) 160-18.

Figure 5 shows the distribution of elastic moduli along the thickness of the sample obtained with 160 °C temperature on the hot cavity side, held for 8 s and 18 s, and evaluated by both indentation and HarmoniX AFM analysis. The distributions compare nicely. This finding confirms the consistency of the results obtained at different scales. Interestingly, the elastic modulus distribution of sample 160-18 moves toward higher values with respect to sample 160-8. In other words, the same morphological structures are characterized by different values of modulus depending on the adopted heating time. When the temperature on the conditioned cavity side is 140 °C (see Figure 3) the elastic modulus in the shear layer on the cold side remains almost similar to the values observed in the shear layers of the CIM sample. A small increase in the modulus values in the shear layer, with respect to the CIM sample, has been observed in the sample obtained with 140 °C temperature on the hot cavity side, held for 18 s. However, this effect is even more pronounced in sample 160-18. This behavior can be ascribed to a more efficient structuring achieved by the polymer chains when high temperature of the conditioned side is maintained for long time [45].

### 3.3. Simulations

**Figure 6:**
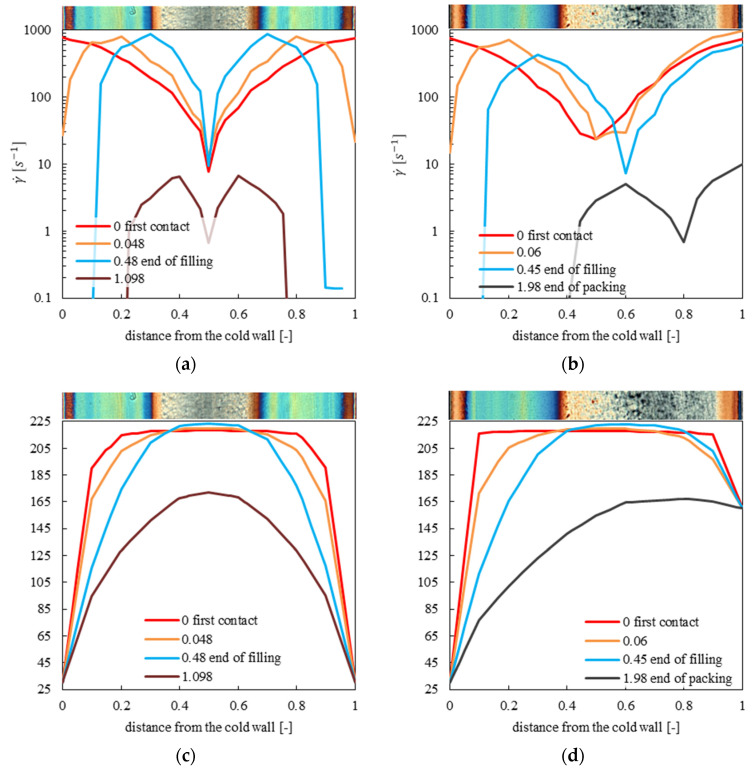
Distributions of shear rate for test CIM (**a**) and 160-8 (**b**); distributions of temperature for test CIM (**c**) and 160-8 (**d**).

Moldflow software (Moldflow 2018, Autodesk Inc., San Rafael, CA, USA) has been adopted for simulating the injection molding process in a temperature conditioned cavity, with the aim of analyzing the shear rate and temperature distributions along the cavity thickness, at the same distance from the gate considered for the mechanical and morphological characterizations. The iPP considered in this work was deeply characterized [46,47] and a Moldflow database material was developed according to that complete characterization. A 3D mesh of the proposed geometry composed of 126,468 tetrahedral elements was adopted. Simulations of tests with cavity temperature modulation were carried out assuming that the temperatures of the tetrahedral elements facing the cavity surface are the same set for the mold surface temperature during the process. The results of the simulations are shown in Figure 6 for CIM and 160-8 tests. In particular, four different time steps were considered, the first contact of the melt with the cavity wall, the end of filling, the end of packing and an intermediate time during filling step.

The shear rate and temperature distributions are symmetrical for the CIM test, whereas the 160-8 test shows asymmetric distributions. Concerning the shear rate distribution, the minimum values of the shear rate are located at the core for the CIM test and it shifts toward the hot cavity side for the 160-8 test. For both tests, the maximum values of the shear rate are located in the shear layer during the filling stage. The maximum values shift toward the cavity center during the duration. Concerning the temperature distribution, the values are close to the melt values during the first-time steps for the majority of the cavity thickness; when the filling ends, the temperature decreases down to the values of the cavity walls; such a decrease is faster close to the cavity walls.

The formation of fibrillar structure depends on both temperature and velocity gradients. High shear rates determine strong molecular orientation. If the cooling rate is so fast as to freeze the orientation of the flow, fibrils can be found in the solid. If the cooling rate is slow, the molecules can relax the stress induced by the flow and fibrils cannot be found in the solid [45,46]. During the filling stage of the CIM test, the cooling rate and the shear rate are high close to the cavity wall, thus, the fibrils form in these regions. Moving toward the cavity core, the cooling rate strongly decreases, and the polymer cannot form fibrils [48], even close to the maximum, and the shear rate remains high. The temperature value in the sample core is close to the injection temperature (220 °C). During the packing stage, the flow intensity (and consequently the shear rate) sharply decreases and cooling occurs at different rates depending on the distance from the cavity wall, in other words, the cooling rate is inversely proportional to the distance from the cavity wall. The comparison between the optical micrograph and calculated shear rate and temperature distributions shows that the fibrils form where the shear rate is higher than 6 s^–1^ and temperature is lower than 160 °C.

**Figure 7:**
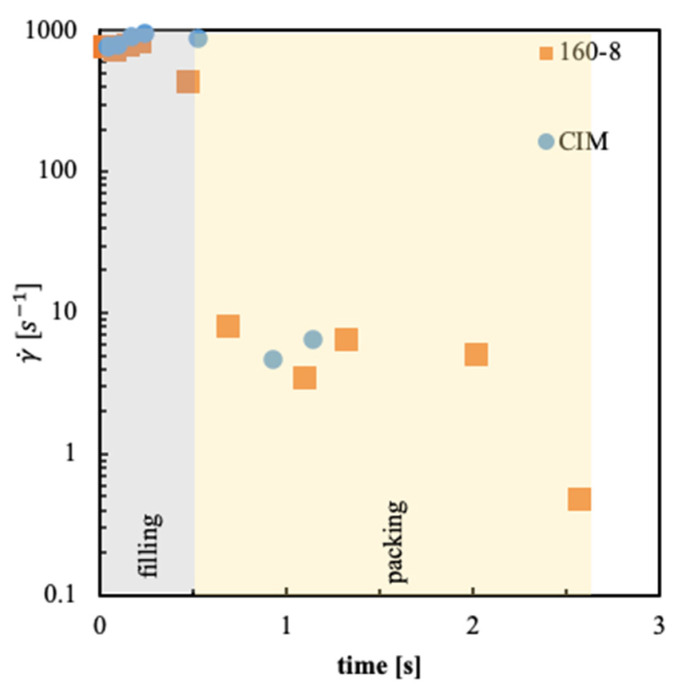
Flow duration in terms of the last time at which the shear rate shows the maximum value.

A better understanding of the different morphologies and mechanical characteristics between 160-8 tests and the CIM test can be obtained considering the evolution of the maximum value of the shear rate for the CIM and 160-8 tests as shown in Figure 7. In particular, the flow necessary for fibril formation is active for a longer time in the 160-8 test.

For sample 160-8 the shear rate is higher than 6 s^–1^ up to 0.4 distance from the cold wall, thus the region characterized by fibrils is larger on the cold side than the region observed in CIM. This behavior depends on the longer flow (about 2 s, see Figure 7) with respect to the CIM case. Such a flow is efficient in the formation of fibrils in a wider region close to the cold side of the cavity than in the CIM test. On the hot cavity side, the temperature is close to the threshold value for fibril formation. In this region, the shear rate is higher than 6 s^–1^ for the time the flow is active and the cooling rates are high. As a consequence, the formation of fibrils can be observed in a thin region close to the hot cavity wall. In the adjacent layers toward the core, the fact that the shear rate is high is not sufficient for fibril formation, since the temperature is higher than the threshold value, therefore, the relaxation times are very small, and the stress can relax very quickly.

High temperature applied for a longer time allows the fibrils to melt. If the cavity side is conditioned for a longer time, 18 s, fibrils disappear in the layers close to the hot cavity side. Indeed, adopting a higher heating time than the relaxation time of the considered iPP at the same temperature (that is lower than 19 s [49]), the molecules can relax the stress accumulated during the flow. While the heating stage lasts, the layers close to the hot cavity side undergo an almost quiescent crystallization and spherulites can be found in the solid.

Test 160-18 shows high temperatures for longer times along the whole thickness with respect to the CIM test. Obviously, the increase of the heating time induces an increase of the time the molecules experience high temperatures. While the molecules experience high temperature for long times, in the temperature range for crystallization, structuring of the molecules occurs. The higher the structuring level, the higher the values of the elastic modulus are [45]. The uneven temperature conditions along the cavity thickness and the time the temperature is held high on one cavity side determine a certain annealing of the molecular chains. The annealing is responsible for the decrease of the amounts of crystalline defects and lamellar thickening. The consequence of lamellar thickening is the increase of the elastic modulus [50]. At temperatures well below melting, a solid-diffusion mechanism, localized to small segment chains, occurs. Under this condition, the orientation is not lost, and fibrils can still be detected [51]. The shear layer on the cold side experiences an annealing at low temperatures, thus, only solid-diffusion mechanisms may occur. As a consequence, one can expect that the fibrillar structures are characterized by higher lamellar thickness than the shear layer of the CIM sample. The higher the temperature, the higher the tendency toward partial melting or recrystallization of the crystalline phase [51,52]. In other words, the diffusion mechanism may involve the whole molecule and partial melting and recrystallization may occur. This is the case of a spherulitical core that experiences temperature closer to melting toward its hot side. Along this cavity thickness, both solid-diffusion and partial melting contribute to the increase of the lamellar thickness; as a consequence, elastic modulus can achieve values close to the values of the shear layer in the CIM sample.

## 4. Conclusions

In this work, injection molding experiments have been carried out with a high non-symmetric heating applied for different durations (0.5, 8 and 18 s) to the one side of the cavity. Resulting samples are mechanically characterized at different length-scales by indentation and HarmoniX AFM.

Asymmetric mold heating, even if applied for a short time, completely modifies the conventional skin-core structure shown by the injection-molded samples. The core layer, characterized by a spherulitic morphology, increases in thickness and moves up to the heated sample skin. On the contrary, the shear layer closer to the heated side moves toward the conditioned side and gradually decreases for 18 s heating duration until completely disappearing. The shear layer originally located closer to the unheated surface increases in dimension and gradually moves toward the core direction. Finally, the resulting skin layer is practically unaffected on the unconditioned side, whereas it completely disappears on the conditioned side. The changes in morphology determine parallel changes in mechanical properties distribution. High mold temperature allows some molecular relaxation with a strong reduction in the elastic modulus in the shear layer close to the heated surface. This effect is not detectable in the opposite half-thickness of the sample where the cooling rate is again sufficient to hold the molecular organization induced by the flow and thus preserve the higher level of moduli.

For the skin layer, despite the heating applied, the simultaneous high molecular orientation and high cooling rate are sufficient to preserve the pristine disordered morphology with relative low modulus. It clearly suggests that to reach high elastic modulus values, it is not sufficient to have high levels of molecular orientation. The samples obtained with the longer heating times show higher values of the elastic modulus with respect to the samples obtained with short heating times, along the whole sample thickness. This finding suggests that polymer molecules experience solid-diffusion and partial melting and recrystallization phenomena during the time they spend under high temperatures. These phenomena allow lamellar thickening with subsequent increase of elastic modulus. Such an increase is more pronounced in the spherulitical layers, where molecules experience temperatures closer to melting, allowing both solid-diffusion and partial melting and recrystallization. In the shear layer of the unconditioned side, the lower temperatures only allow the solid-diffusion phenomenon, thus the lamellar thickening, and the increase of the elastic modulus, is smaller with respect to the ones that have to occured in the spherulitical layer.

## Figures and Tables

**Table 1:** Experimental molding conditions.

Parameters	Values
injection temperature	220 °C
injection time	3 s
flow rate	4 cm^3^ s^–1^
packing pressure	300 bar
packing time	2 s
cavity temperature, cold side	25 °C
cavity temperature, hot side	25 °C (CIM), 140 °C, 160 °C
heating time, t_h_	0 (CIM), 0.5, 8 and 18 s
